# Supplementary material for: Intermolecular Interactions in 3-Aminopropyltrimethoxysilane, N-Methyl-3-aminopropyltrimethoxysilane and 3-Aminopropyltriethoxysilane: Insights from Computational Spectroscopy
Source: Int J Mol Sci. 2023 Nov 23;24(23):16634. doi: 10.3390/ijms242316634 (PMC10706811; doi:10.3390/ijms242316634)
Supplement: Supplementary file 1 [file ijms-24-16634-s001.zip › ijms-2717430-supplementary.pdf]

Intermolecular interactions in 3-aminopropyltrimethoxysilane , N-methyl-3-aminopropyltrimethoxysilane and 3-aminopropyltriethoxysilane: Insights from computational spectroscopy

Mariela M. Nolasco[1*], Stewart F. Parker[2], Pedro D. Vaz[3], and Paulo Ribeiro-Claro[1]

[1] CICECO – Instituto de Materiais de Aveiro, Departamento de Química, Universidade de Aveiro, P-3810-193 Aveiro, Portugal

[2] ISIS Neutron & Muon Source, STFC Rutherford Appleton Laboratory, Chilton, Didcot, Oxfordshire OX11 0QX, UK

[3] Champalimaud Foundation, Champalimaud Centre for the Unknown, 1400-038 Lisboa, Portugal

Electronic Supplementary Material

Contents:

Table S1– Calculated energies of the relevant optimized structures

Figure S1 – INS spectra of APTS, MAPTS, and APTES up to 4000 cm^{-1}.

Figure S2 – Calculated infrared spectra of MAPTS

Figure S3 – Calculated Raman spectra of MAPTS

Figure S4 – Experimental and calculated infrared spectra of APTES.

Figure S5 – Experimental and calculated Raman spectra of APTES.

Table S1– Calculated energies of the relevant optimized structures.

Electronic energies at the M062X /6-311+g(d,p) level, without basis set superposition error (BSSE) and zero-point vibrational energy correction (ZVPE).

Dimerization energy = Dimer energy – 2x(Monomer energy); Trimerization energy = Trimer energy – 3x(Monomer energy).

	**Total Energy** (Hartree)	**Dimerization Energy** (kJ/mol)	**Trimerization Energy** (kJ/mol)
**APTS**			
Monomer	-808.820558775		
Dimer HH	-1617.654579760	-35.3	
Dimer HT	-1617.654539840	-35.2	
Trimer HH	-2426.488339430		-70.0
Trimer HT	-2426.495122090		-87.8
**MAPTS**			
Monomer	-848.114986059		
Dimer HH	-1696.244926760	-39.3	
Dimer HT	-1696.245676290	-41.2	
Trimer HH	-2544.374679090		-78.0
Trimer HT	-2544.383062290		-100.0
**APTES**			
Monomer	-926.74584363		
Dimer HH	-1853.50781342	-42.3	
Dimer HT	-1853.50995021	-47.9	
Trimer HH	-2780.26976464		-84.6
Trimer HT	-2780.27991486		-111.3

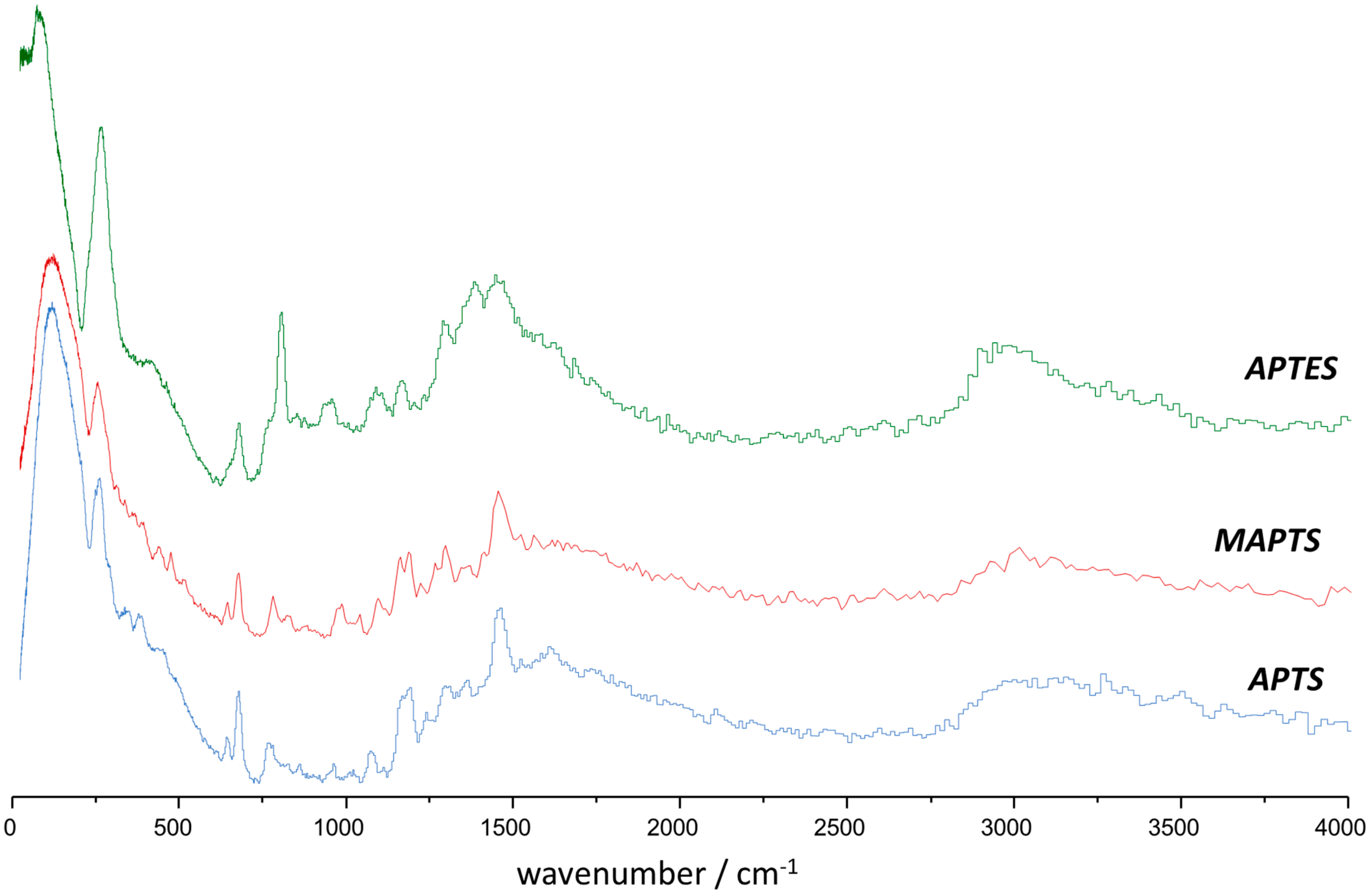

Figure S1. *INS spectra of APTS, MAPTS, and APTES up to 4000 cm^{-1}.*

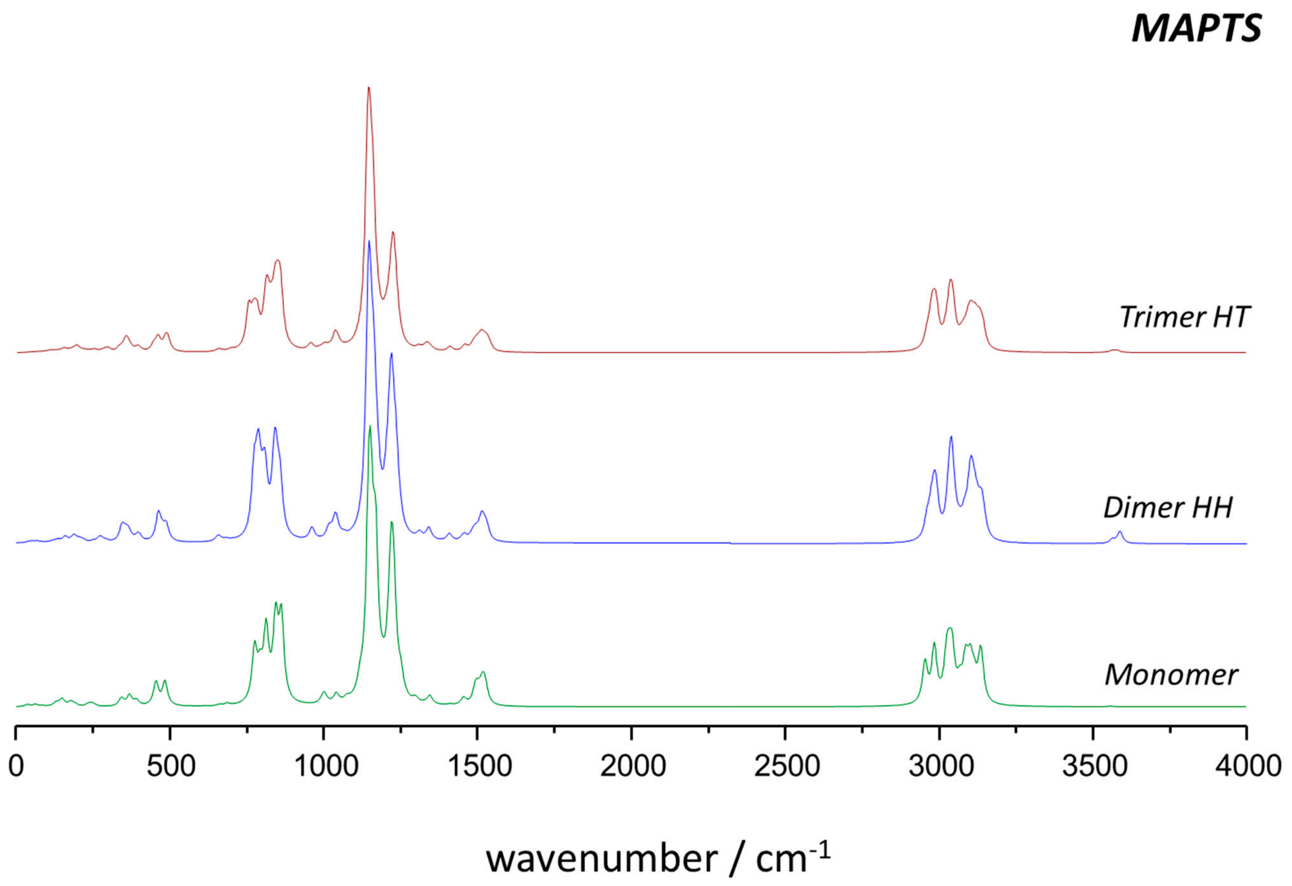

Figure S2. *Calculated infrared spectra of MAPTS, up to 4000 cm^{-1}. Calculated spectra in coloured lines, from bottom: monomer (green), head-to-head dimer (blue), head-to-tail trimer (red).*

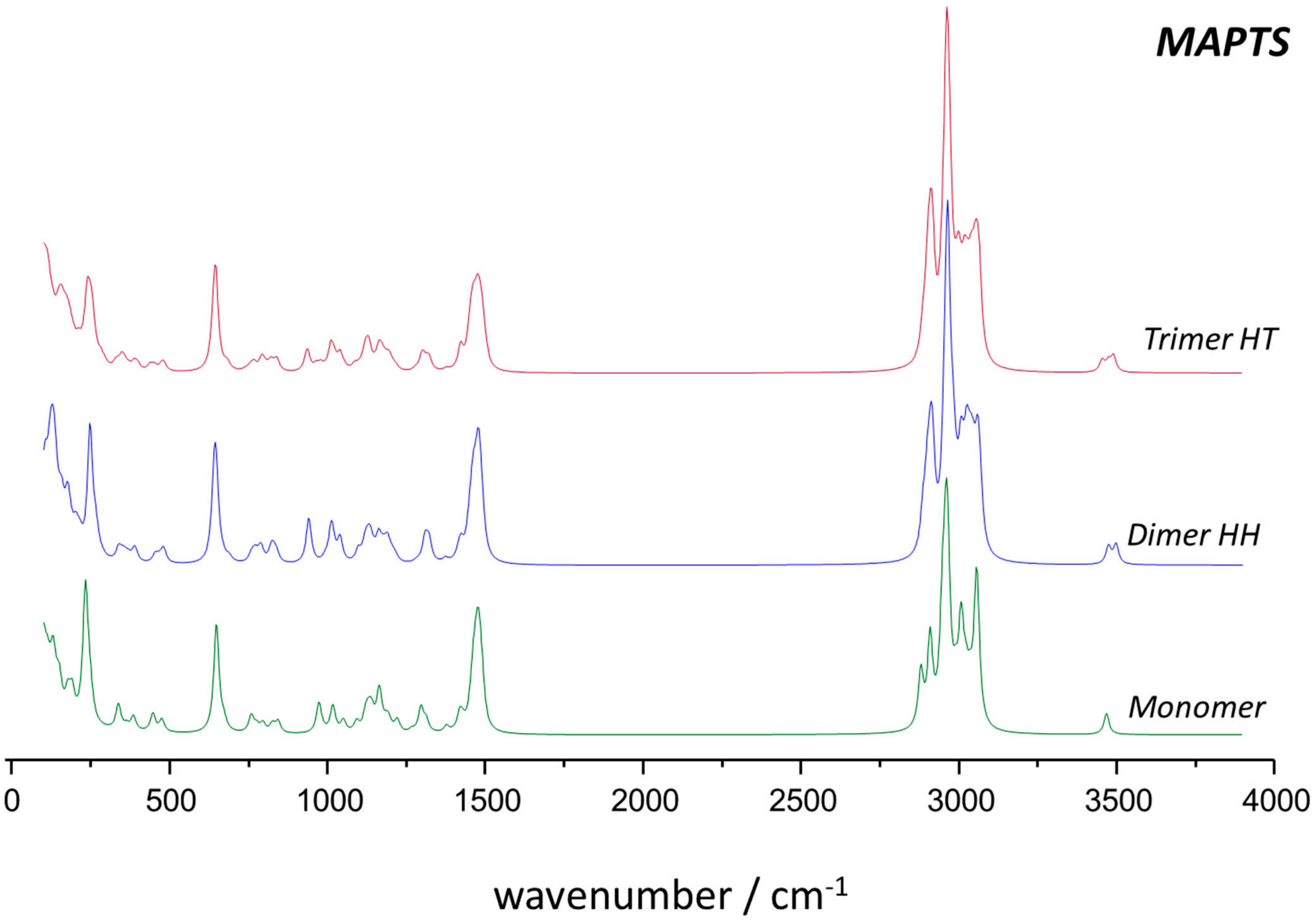

Figure S3. *Calculated Raman spectra of MAPTS, up to 4000 cm^{-1}. Calculated spectra in coloured lines, from bottom: monomer (green), head-to-head dimer (blue), head-to-tail trimer (red).*

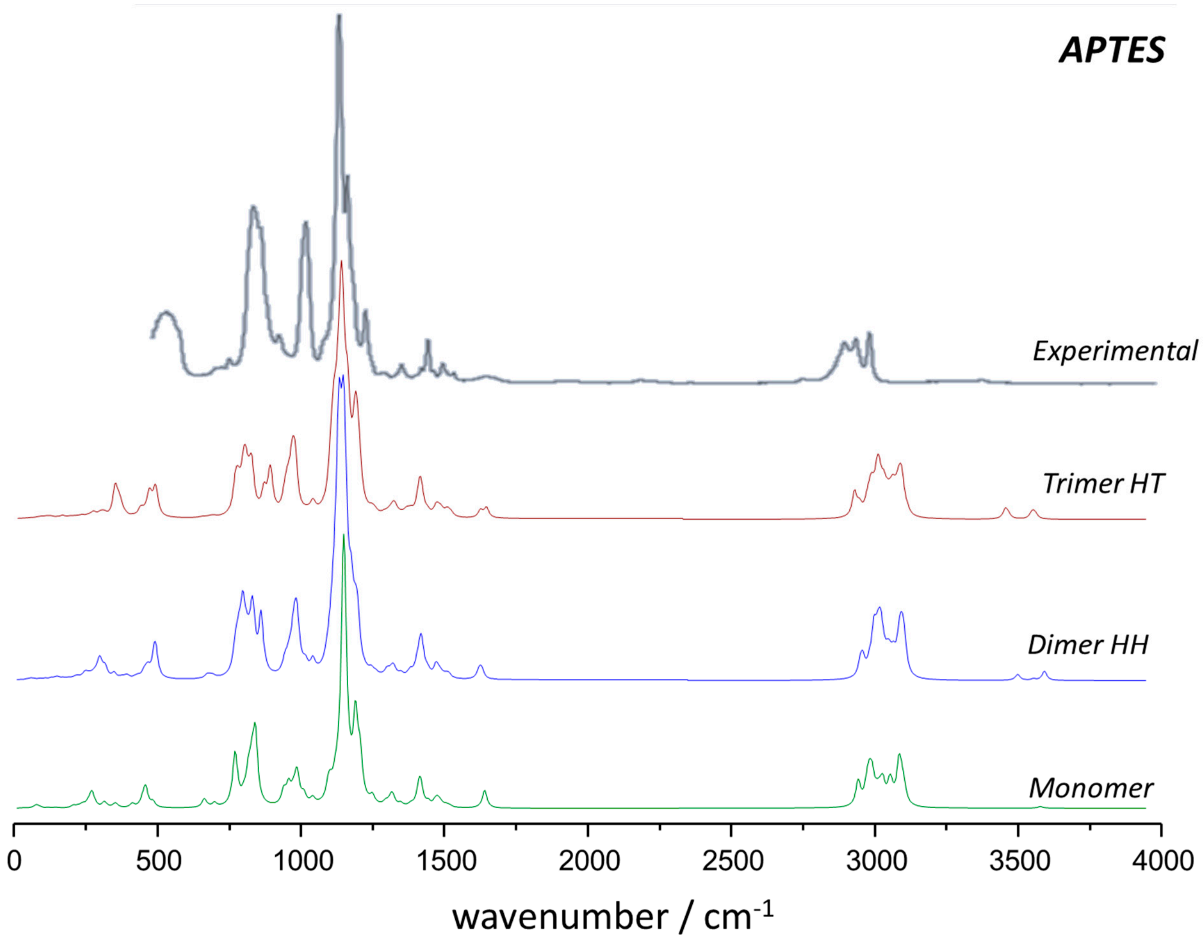

Figure S4. *Experimental and calculated infrared spectra of APTES. Top line: experimental spectrum, up to 4000 cm*$^{-1}$*. Calculated spectra in coloured lines, from bottom: monomer (green), head-to-head dimer (blue), head-to-tail trimer (red).*

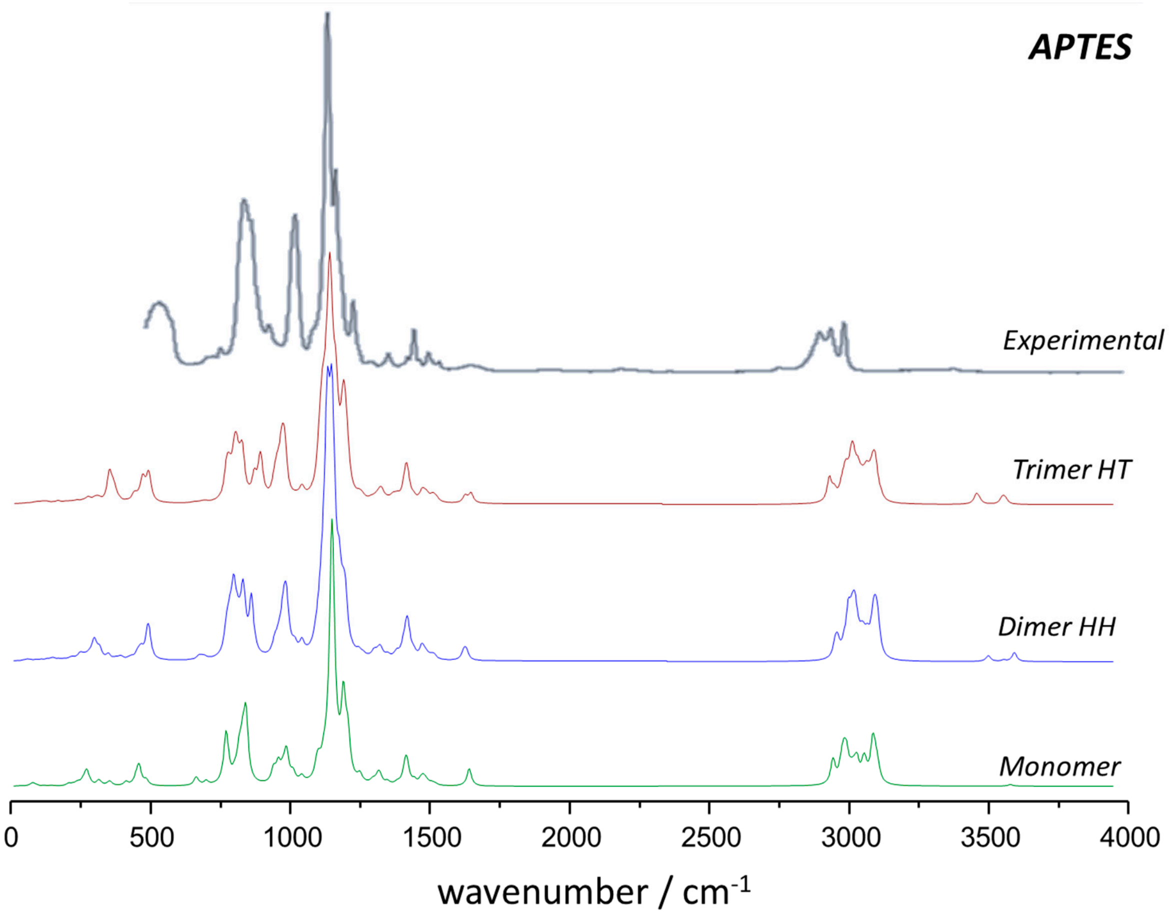

Figure S5. *Experimental and calculated infrared spectra of APTES. Top line: experimental spectrum, up to 4000 cm^{-1}. Calculated spectra in coloured lines, from bottom: monomer (green), head-to-head dimer (blue), head-to-tail trimer (red).*
